# Basics of Antibody Phage Display Technology

**DOI:** 10.3390/toxins10060236

**Published:** 2018-06-09

**Authors:** Line Ledsgaard, Mogens Kilstrup, Aneesh Karatt-Vellatt, John McCafferty, Andreas H. Laustsen

**Affiliations:** 1 Department of Biotechnology and Biomedicine, Technical University of Denmark, Kongens Lyngby DK-2800, Denmark; Lineledsgaard@hotmail.com (L.L.); mki@bio.dtu.dk (M.K.); 2 IONTAS Ltd., Cambridgeshire CB22 3EG, United Kingdom; akv@iontas.co.uk (A.K.-V.); jmc@iontas.co.uk (J.M.)

**Keywords:** antibody discovery, recombinant antivenom, phage display, M13 phage, toxinology

## Abstract

Antibody discovery has become increasingly important in almost all areas of modern medicine. Different antibody discovery approaches exist, but one that has gained increasing interest in the field of toxinology and antivenom research is phage display technology. In this review, the lifecycle of the M13 phage and the basics of phage display technology are presented together with important factors influencing the success rates of phage display experiments. Moreover, the pros and cons of different antigen display methods and the use of naïve versus immunized phage display antibody libraries is discussed, and selected examples from the field of antivenom research are highlighted. This review thus provides in-depth knowledge on the principles and use of phage display technology with a special focus on discovery of antibodies that target animal toxins.

## 1. Introduction

With the recent inclusion of snakebite envenoming on the World Health Organization’s list of Neglected Tropical Diseases [1], focus on both prevention and treatment of this infliction has increased. This creates renewed hope for snakebite victims worldwide and could potentially lead to a mobilization of scientific efforts toward the development of novel snakebite envenoming therapies. Several different avenues aimed at bringing innovation into the field of snakebite antivenoms have been pursued, including medicinal chemistry approaches, novel immunization techniques, and the use of biotechnological strategies [2,3,4,5]. One promising approach seems to be the use of human IgG antibodies [6] and/or camelid antibody fragments [7,8], as these molecules can be used to develop recombinant antivenoms with high efficacy and safety due to their compatibility with the human immune system [2]. Moreover, these therapeutic proteins could be manufactured cost-competitively using modern cell cultivation methods employed for large scale production [6,9]. To discover and develop antibodies, different techniques can be harnessed. One approach is phage display selection [10], which is a robust, easy-to-perform, and inexpensive method by which specific antigen binders are selected from large combinatorial libraries containing billions of antibody fragments.

As antibody phage display is gaining increasing interest in the field of toxinology, the intention with this review is to provide both basic and more advanced knowledge on the underlying science behind the technology and the lifecycle of the M13 phage.

## 2. The M13 Bacteriophage

Central to phage display technology is the biology of the bacteriophage used to display antibodies. Different bacteriophage systems can be utilized for phage display, including the T4, lambda, as well as the filamentous M13 bacteriophage [11]. These different phage systems each have their benefits and drawbacks. However, primarily the M13 phage has been utilized extensively in recent times, and to the best of our knowledge, this is the only phage system that has been explored within toxinology [2,12]. Therefore, this review will focus on antibody phage display techniques utilizing this specific phage system. Before giving an in-depth description of the steps involved in phage display experiments, an introduction to the wild-type M13 phage is provided.

**Figure 1:**
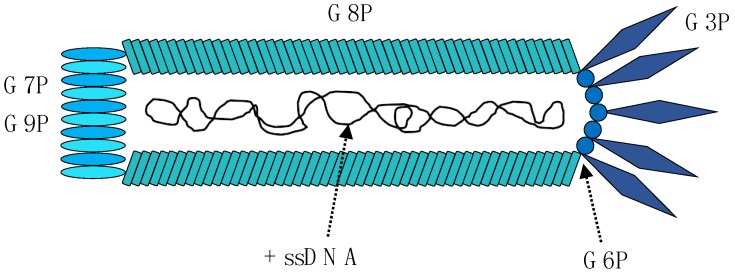
Schematic representation of the M13 bacteriophage, which is a filamentous phage carrying a single-stranded DNA (ssDNA) chromosome. The genome contains nine genes, which encode 11 proteins. Five of these proteins are coat proteins (G3P, G6P, G7P, G8P and G9P), while the remaining six proteins are used for replication of the genome, assembly of the phage, and phage extrusion.

The M13 phage [13] belongs to a group of filamentous phages collectively referred to as Ff phages [14]. The Ff phages only infect *Escherichia coli* strains that express the F pilus as the adsorption of the phage to the bacterium requires binding of a phage coat protein to the tip of the F pilus [15]. The M13 phage is neither temperate nor lytic. Instead, the phage establishes a chronic infection in its host, where it continuously releases new phages. The phage contains a genome of single-stranded DNA (ssDNA) with a length of 6407 bp [16] that consists of nine genes encoding 11 different proteins. Five of these proteins are coat proteins, and the remaining six proteins are involved in replication and assembly of the phage. The M13 phage has a length of 900 nm and a width of 6.5 nm [17]. The most abundant of the coat proteins is the capsid protein G8P, which forms an envelope around the chromosome consisting of approximately 2700 protein units (Figure 1). The remaining four coat proteins, G3P, G6P, G7P, and G9P, are each present in approximately five copies. Information on the genes and proteins of the M13 phage is listed in Table 1.

**Figure 2:**
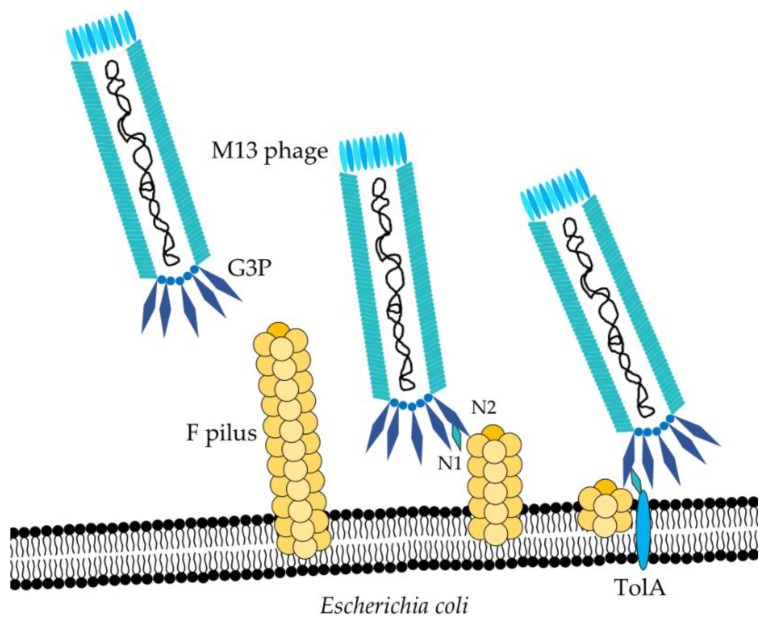
Infection of *Escherichia coli* by the M13 phage. The phage G3P binds to the tip of the F pilus on *E. coli*. Normal disassembly of the F pilus transports the phage to the surface of the bacterium, where it interacts with the TolA receptor, mediating uptake of the phage genome.

The first stage of the M13 infection is the adsorption process, which takes place through binding of the N2 domain of the G3P coat protein to the tip of a F pilus on the surface of *E. coli* hosts (Figure 2) [18,19,20]. Under normal conditions, “male” *E. coli* cells (F^+^, containing F-pili) appear to trawl the environment in an effort to catch “female” (F^−^) *E. coli* partners by a series of F-pilus assembly and disassembly events [21]. Upon phage binding to the tip of one of these F pili, disassembly will automatically bring the phage closer to the surface of the bacterium [18]. Contact between the G3P-N2 domain and the F pilus mediates the allocation of the G3P-N1 domain and allows it to bind TolA, which then functions as a co-receptor on the surface of the bacterium. Three Tol proteins are present in *E. coli*; TolA, TolR, and TolQ, and all are essential for infection by mediating depolymerization of the phage coat and translocation of the ssDNA into the bacterium [20,22,23,24,25].

**Figure 3:**
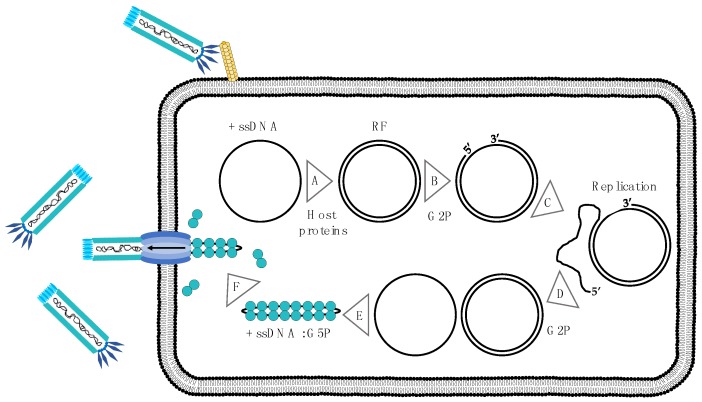
Infection cycle of the M13 phage. (**A**) Upon infection, the single stranded (+) chromosome of the M13 phage is converted into the double-stranded replicative form (RF) (**B**) After proper accumulation, the G2P nicks the (+) strand in the RF and binds covalently to the 5′-end (**C**) The genome is then replicated from the 3′ end of the nick, using the (−) strand as a template. The original G2P-bound (+) strand is physically displaced by the Rep helicase throughout the elongation process (**D**) The old (+) strand is re-circularized by the bound G2P after dissociation, ready to be converted into an RF or to be packaged into new M13 phages (**D**) Genome replication continues until the concentration of G5P has accumulated to sufficient levels to sequester the ssDNA (**E**) When sufficient G5P is accumulated, G5Ps will bind the ssDNA in a back-to-back dimeric conformation, causing the more rod-shaped appearance of the ssDNA (**F**) A pore is formed in the membrane, and the phage genome is translocated through this pore, while the phage coat is assembled.

Once the chromosome of the phage has been injected into the bacterium, the host DNA synthesis machinery will synthesize a DNA (−) strand complementary to the ssDNA (+) strand of the M13 phage. The two strands form a supercoiled double-stranded DNA (dsDNA) phage chromosome, termed the replicative form (RF) (Figure 3A) [26]. Replication of the M13 chromosome always uses the RF and is carried out by continuous rolling circle replication. Initiation of replication starts when G2P has accumulated to a concentration that allows it to nick the (+) strand in the RF and bind covalently to the 5′ end (Figure 3B). The 3′ end that is generated from the nick will then be elongated by DNA polymerase using the (−) strand as a template. Throughout the elongation process, the original G2P-bound (+) strand is physically displaced by the Rep helicase [27,28] (Figure 3C). When one round of replication is completed, the old (+) strand is cut off at the origin by a new G2P, which remains bound to the new 5′ end. After dissociation from the RF, the old (+) strand is re-circularized, ready to be converted into an RF or to be packaged into new M13 phages (Figure 3D). In the early stages of infection, all newly formed ssDNA (+) chromosomes will be converted into RFs, but in later infection stages, where the concentration of G5P is sufficient for fast sequestering of (+) strands, formation of RFs will be prevented. When the G5P binds the ssDNA, it dimerizes in a back-to-back conformation leading to the conversion from the circular appearance of the ssDNA to a more rod-shaped appearance (Figure 3E) [29]. The entire phage chromosome is covered by the G5P except for an exposed hairpin loop termed the packing signal [30]. The packing signal is required for packing of the phage genome. G10P plays an essential but unknown role for the stable accumulation of ssDNA (+) strands. The ssDNA bound by the G5P protein is the substrate for phage assembly (Figure 3F).

Assembly and budding of the M13 phage is a five-step process that includes: preinitiation, initiation, elongation, pretermination, and termination. During preinitiation, an assembly complex is formed, consisting of G1P, G11P, and G4P, all interacting through their periplasmic domains. A cylindrical structure, consisting of 12-14 G4P monomers, mediates close contact between the cytoplasmic membrane and the outer membrane. In addition to a large structure generated by G4P, a multimeric complex containing five to six copies of both G1P and G10P is generated, potentially forming a second channel [22].

Initiation awaits formation of the assembly complex during preinitiation as well as accumulation of G5P-bound M13 chromosomes, and subsequently positions G7P and G9P at the tip of the complex. Here, G7P and G9P interact with the packing signal in the phage chromosome, which facilitates contact with the G1P, resulting in binding of thioredoxin from the cell [31].

During the elongation step, the G5P bound to the phage genome is replaced with G8P for translocation of the DNA through the membrane-spanning channel (Figure 3F). The translocation continues until the phage genome has become completely coated with G8P, at which point G3P and G6P will collaborate in the release of the phage from the bacterium. If either G3P or G6P is missing, additional phage genomes can be loaded through the pore resulting in formation of a much longer phage particle [32,33].

During pretermination, membrane-embedded G3Ps complexed with G6Ps are incorporated at the terminal end of the phage particle. Termination involves the release of the phage, brought about by a conformational change in the G3P-G6P complex [32,34].

## 3. Using the M13 Phage as a Tool in Antibody Discovery

**Figure 4:**
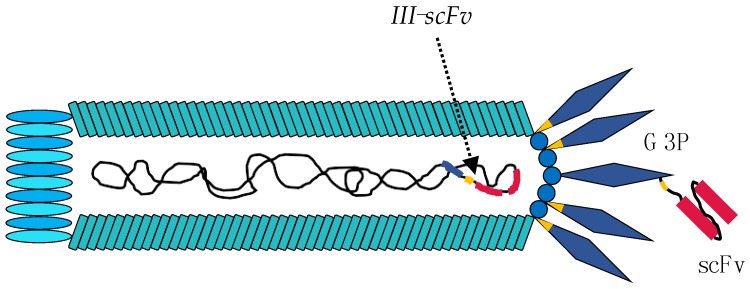
Engineering of the M13 phage for phage display experiments. The G3P is genetically fused to a human single-chain variable fragment (scFv) with a trypsin cleavage site shown in yellow as part of a peptide linker connecting the two proteins.

Phage display technology was demonstrated in 1985 by Smith, who successfully incorporated foreign DNA into the M13 phage chromosome such that foreign peptides were fused to the G3P coat protein of the M13 phage [35]. Five years later, antibody phage display selection was first described by McCafferty et al., who were able to fuse genes encoding an entire antibody binding domain (in the form of single-chain variable fragments, scFvs) to gene *III* (Figure 4) [36]. This approach facilitated a sufficient level of antibody display on the outer surface of the phage virion to permit selection of antigen-recognizing phages [36]. The method thus exploits the possibility of directly linking a protein (phenotype) to its cognate gene (genotype) through a phage. Since 1990, different antibody formats have been employed in the construction of antibody-displaying phage libraries, including heavy-domain human antibody fragments (V_H_s), heavy-domain camelid and shark antibody fragments (V_H_Hs), scFvs, diabodies (bivalent scFvs), and entire fragments antigen binding (Fab) antibodies [37,38,39,40]. Generally, these antibody fragments are fused to the G3P of the M13 phage (Figure 4), and by cloning large numbers of genes encoding an antibody fragment, large phage display antibody libraries can be generated from which many diverse antibodies can be selected. As an example, Schofield et al. successfully selected more than 7200 recombinant antibodies to 292 antigens from a human scFv library containing 1.1 × 10^10^ clones [41].

## 4. Phage and Phagemid Libraries

In the earliest examples, antibody genes were cloned directly into the filamentous phage genome, which carries all the genes needed for infection, replication, assembly, and budding while also carrying the gene encoding the antibody-G3P fusion. Since each phage particle normally incorporates three to five copies of G3P, the use of phage vectors potentially results in a multivalent display of antibody-G3P fusion proteins (although proteolytic degradation can result in removal of a proportion of the fused antibody). As an alternative, phagemid vectors based on smaller “minimal plasmids” may be used. Phagemid vectors contain three key elements (i) an antibiotic marker for selection and propagation of the plasmid (ii) the gene encoding the antibody-G3P fusion protein, and (iii) the regions of the M13 chromosome (phage origin of replication) required for rolling circle amplification and the production of the (+) DNA strand that is capable of being packaged into a phage. In order to produce functional phage particles displaying antibody-G3P fusion, *E. coli* harboring a phagemid vector is infected with a “helper phage”. The helper phage contains the complete M13 genome encoding all the phage proteins needed for capsid production, phage assembly, chromosome replication, and budding (the concept of helper phage is described in Section 5 in detail). Upon infection, wild-type G3P from the helper phage competes with the phagemid encoding the G3P-scFv fusion protein for incorporation into the phage. Ninety percent of the resulting phage population do not display any fusion protein and the vast majority of the phage particles that bear the fusion protein will only contain a single copy [42]. Phagemid vectors are more typically used in library construction than phage vectors because higher transformation efficiencies can be achieved facilitating the construction of larger libraries. In a direct comparison of libraries created using phage versus phagemid vectors, it was shown that a greater diversity of antibody binders was generated from libraries built using the phage vectors rather than phagemid vectors. The phage system allows for the emergence of a wider range of antibody affinities compared to the phagemid system [43]. Although the phage system is often referred to as a polyvalent display and the phagemid system as monovalent, in practice degradation occurs in both cases, and the beneficial effects of a phage vector-based system may also be attributed to the presence of a higher proportion of non-bald phage particles. Methods have been developed to restore antibody display levels in phagemid libraries to the same levels achieved in phage vector-based systems. This is based on the use of helper phages, which do not encode G3P (the so called hyperphage system [44]), so that only the G3P-scFv encoded by the phagemid is available for presentation. Since G3P is needed for packaging and infection of the helper phage and the hyperphage genome lacks the gene encoding G3P, it is necessary to provide this in trans by using bacteria which express G3P from within the bacterial genome.

## 5. Phagemid Libraries Are Amplified Using Helper Phages

**Figure 5:**
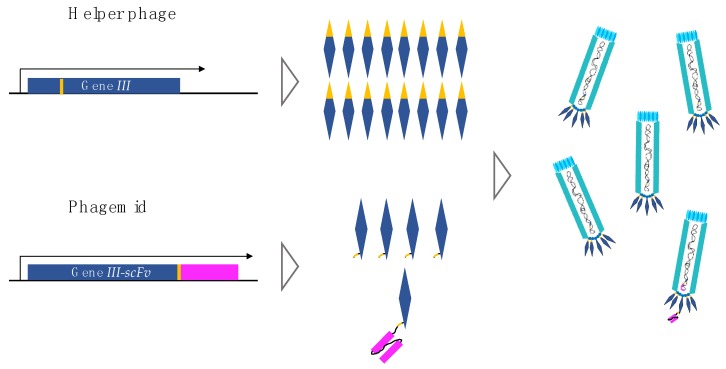
Gene *III* product from helper phage and gene *III-scFv* product from phagemid. The helper phage carries the gene encoding a trypsin-sensitive ‘bald’ G3P. The phagemid carries the gene encoding the G3P-scFv fusion protein. As M13 phages will be assembled with three to five copies of G3P, the number of assembled phages containing more than one G3P-scFv fusion protein will be too low to influence the outcome of selections, because more ‘bald’ G3P than G3P-scFv fusion proteins will be expressed. Thereby, the majority of the scFv-displaying phagemids will only be carrying one scFv (monovalent display).

As mentioned earlier, an M13 helper phage is used for packaging of phagemid particles in *E. coli*. The helper phage carries all genes necessary for infection, replication, assembly, and budding and therefore provides the phagemid, which primarily carries the gene encoding the G3P-scFv fusion protein, with the proteins needed for amplification. One such helper phage is the M13KO7 phage [45]. This helper phage carries a heterologous, low copy origin of replication from the plasmid p15A inserted within the native M13 origin of replication. When the M13KO7 phage is present alone in a host bacterium (i.e., during preparation of helper phage), the replication is sufficient for producing high titers of M13KO7 phage. However, if a high copy number phagemid with an intact M13 origin is present, this will out-compete the helper plasmid during packaging, meaning that the majority of resulting phage particles will carry phagemid DNA [45]. The resultant phagemid particles will incorporate G3P encoded by the helper phage genome as well as G3P-scFv encoded within the packaged phagemid. In practice, there is preferential incorporation of the wild-type G3P from the helper plasmid, meaning that the majority of the phagemids will be ‘bald’ (not displaying an antibody fragment). The preferential presentation of G3P rather than the G3P-scFv fusion is probably due to a combination of differences in expression/translation levels and the fact that a proportion of the G3P-scFv fusion is degraded (Figure 5). An overview of which genetic elements are carried by the phagemid and the helper phage is provided in Table 2.

**Figure 6:**
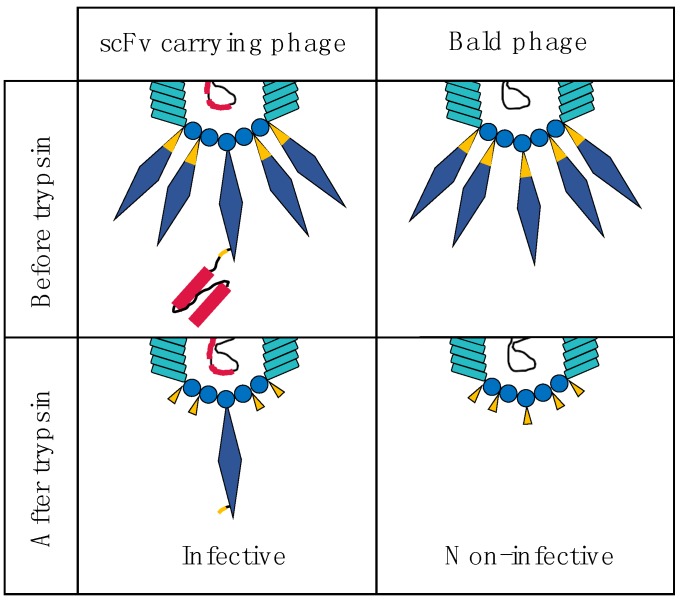
Trypsin treatment of G3P and G3P-scFv fusion proteins on the M13 phage. G3P encoded by the helper phage is trypsin-sensitive and can thus be cleaved by trypsin, rendering the phage non-infective. In contrast, the G3P-scFv fusion protein encoded by the phagemid contains a myc-tag in the linker between the G3P and the scFv, which will be cleaved during trypsin treatment. This leaves a G3P on the phage, rendering it infective.

Bald phages do not participate in positive antibody: antigen selection and only contribute to background noise within the system. It is possible to reduce the background noise from this population by rendering them non-infectious. This is achieved by engineering gene *III* within the helper phage to render the encoded G3P sensitive to trypsin [46]. Trypsin treatment before infection will result in inactivation of bald phages, which rely on labile G3P encoded by the helper phage (Figure 6). In contrast, phages carrying a G3P-scFv fusion, encoded by the phagemid, will be resistant to G3P disruption and will retain infectivity after trypsin treatment (As previously shown in Figure 4, the G3P-scFv fusion protein encoded by the phagemid contains a trypsin cleavage site, but this is situated between the scFv and the G3P, and thereby does not prevent infection (Figure 6)).

## 6. Performing a Phage Display Selection Experiment

**Figure 7:**
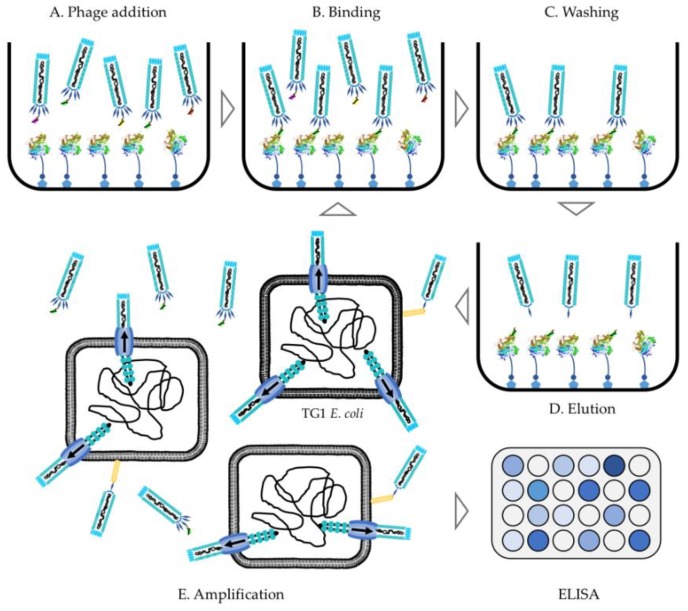
The five steps in a phage display selection experiment. Addition of phage library (**A**) refers to the addition of phages to an antigen-coated vial. The phages displaying the highest affinity scFvs will bind (**B**) the antigen, while unspecific binders will be removed during the washing step (**C**).After washing, the antigen-specific phages can be eluted (**D**) using trypsin digestion (or other means of elution) Then, phages are amplified (**E**) in *E. coli* and a new panning round can be initiated to further accumulate phages displaying high affinity antibody fragments. Binding can be evaluated using both ELISA and plate tests.

Phage display selection is a high throughput method used to discover antibodies specific to different antigens. The protocol includes rounds of five steps (Figure 7). The first step is addition of a phage library, where the library is added to a well or vial in which the antigen is presented (Figure 7A). Antigen presentation can be achieved via direct coating (adsorption) or via a capture system (such as streptavidin-biotin). The second step involves binding, where the phages displaying the highest affinity antibodies bind the epitopes of the antigen (Figure 7B), after which the vial is washed to remove non-binding phages (Figure 7C). After washing, the bound phages are eluted by enzymatic digestion using trypsin or by other means of elution (e.g., dilute acid or base) (Figure 7D). When enzymatic digestion is employed, this step renders bald phages non-infective [46] (Figure 6), thereby improving the selection of antibody-displaying phages that are still infective (see previous section). In the fifth step, helper phages are added to allow for amplification of the eluted infective phages in *E. coli* (Figure 7E). To accumulate phages displaying high affinity antibody fragments, these five steps are usually repeated 1-3 times with the amplified phages from the preceding round of panning. Titration of phage output numbers may also be utilized to monitor progress, and polyclonal phage ELISA used to confirm selection of a population of binders.

Selections can also be performed in solution with a subsequent pull-down step to isolate high affinity scFv-displaying phages that are bound to the antigen (which typically needs a tag that can be pulled down, such as a biotin moiety) [47]. This may also serve to avoid antigen immobilization via hydrophobic regions, which may diminish the accessibility of relevant epitopes as discussed below. Conceptually, selections performed in solution include the exact same steps with a few practical changes. Selections in solution may be beneficial for certain antigens, which better retain proper folding and conformation that resembles the native antigen protein. Possibly, this is particularly useful for antigens that natively exist in solution (e.g., blood factors, toxins, cytokines, and hormones).

## 7. Antigen Quality and Presentation

**Figure 8:**
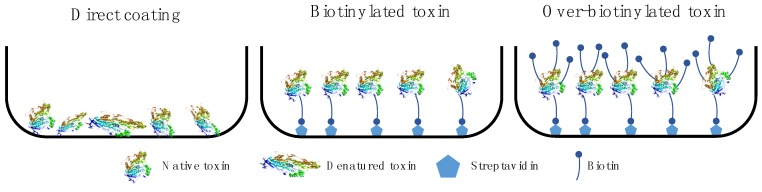
Antigen immobilization strategies. Direct coating can result in denaturation of the antigen or distortion of its conformation, whereas immobilization through a streptavidin-biotin capture system, involving biotinylation of the antigen, can give rise to over-biotinylation resulting in low availability of binding sites.

In addition to having a phage display library of high quality, the most crucial element for the success of a phage display selection experiment is antigen presentation. Different methods of antigen presentation exist with different advantages and disadvantages (Figure 8) [48,49]. The simplest method of antigen presentation is to coat the antigen directly to the plastic surface of a well or vial via adsorption. The major disadvantage of direct immobilization is that the antigen might undergo conformational changes upon binding to the well or vial surface, potentially leading to conformational distortion or denaturation of the antigen. Selection of antibodies against a distorted or denatured antigen will result in accumulation of phages displaying antibodies with high affinity to the distorted antigen but low affinity to the native antigen in its proper conformation. Additionally, antigens might have preferences regarding how they bind to the surface, which might result in areas of the antigen not being properly exposed for interaction. Therefore, very few phages displaying antibodies with high affinity to such an area can be selected, which is problematic if this area is important to target for antigen neutralization.

Antigen presentation by immobilization through a streptavidin-biotin capture system requires that the antigen is chemically conjugated to a biotin moiety via a linker. Biotinylation of an antigen can be a challenging task, especially since the biotin-to-antigen ratio can be crucial but difficult to control. Over-biotinylation of the antigen may heavily decrease the available surface area on the antigen, thereby preventing phage binding. Also, over-biotinylation may alter the physico-chemical properties of the antigen, possibly leading to undesirable effects, such as antigen aggregation. The main advantage of immobilization through a biotin linker is that the antigen will be lifted from the plastic surface of the vial bottom by first coating the vial with streptavidin (or neutravidin) and then adding the biotinylated antigen to be captured. This allows for an improved display of the antigen and thus the selection of phages displaying relevant antibodies against more antigen epitopes [48]. A disadvantage of immobilization through a biotin linker is that addition of streptavidin (or neutravidin) introduces an additional macromolecule that the phage can bind to, which may lead to accumulation of phages with streptavidin/neutravidin-binding antibodies. To avoid this issue, deselection steps must be implemented in the phage display protocol when working with antigens that are immobilized using such capture systems.

Regarding optimal antigen display via capture systems, it can be worthwhile to consider which coupling chemistry should be employed. The benefit of having a site-specific coupling reagent includes greater control; however, this may in some cases have a negative interference with a potential binding site, which may lead to poor selection. As an example, site-specific coupling to the N-terminal is likely to be suboptimal if the N-terminal region is part of an important epitope-paratope interaction.

## 8. Naïve versus Immunized Phage Display Antibody Libraries

Phage display antibody libraries can be derived from either non-immunized (naïve) or immunized donors depending on whether the donors, from which the antibody genes were isolated and used to create the library, have been immunized with an antigen or not. The benefit of naïve libraries (often from the IgM repertoire from non-immunized donors) is that it can be used for discovery of a wide range of antigens, although the discovered antibody fragments may generally have lower affinities than antibodies discovered from an immunized source [7,50]. In a phage display antibody library from an immunized source (from the IgG repertoire), the antibody fragments have increased affinity towards the type of antigen used for immunization as the antibodies have gone through an in vivo affinity maturation process. This can be an advantage in antibody discovery as it will more easily generate high affinity hits. Antibodies from the IgG pool of immunized sources will generally have less diversity than naïve IgM-based libraries, and they are therefore not as broadly applicable in terms of their antigen scope.

**Figure 9:**
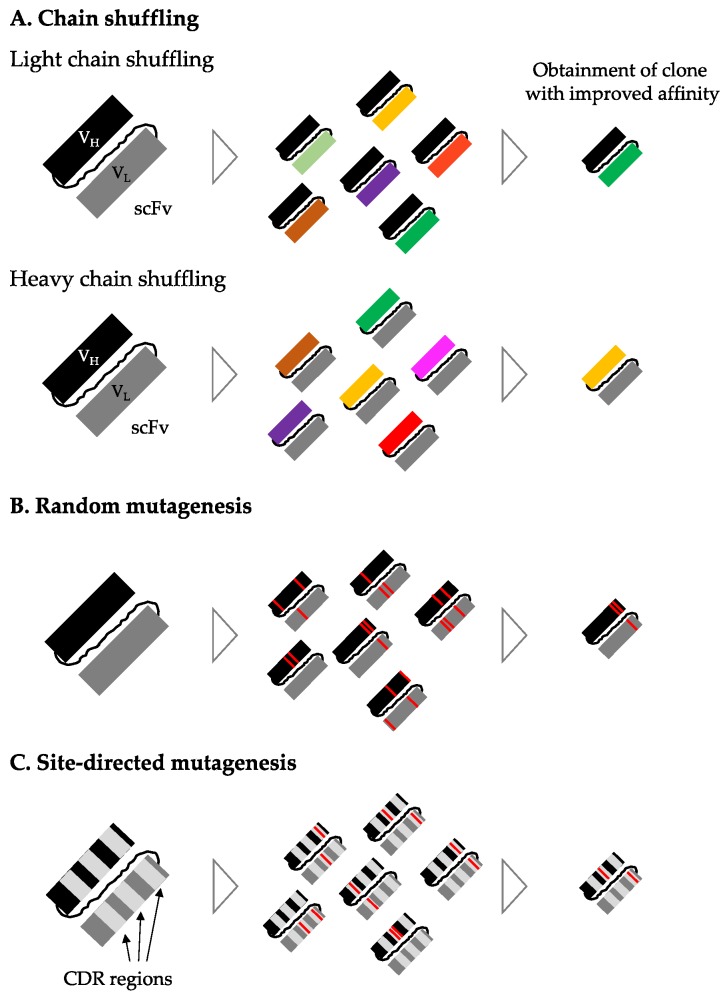
Diversification methods used for in vitro affinity maturation of antibody fragments. Chain shuffling (**A**) is where one heavy or light chain is paired with chains of the opposite type from a naïve library. The principle of both light and heavy chain shuffling is presented in the figure. Random mutagenesis (**B**) is used to introduce mutations (red lines) in the entire variable regions of the antibody fragment. Site-directed mutagenesis (**C**) is used to specifically introduce mutations in one or more of the complementarity-determining regions (CDR) regions of the antibody fragment.

Different techniques can be employed for in vitro affinity maturation of antibody fragments from naïve phage libraries. The basic principle of in vitro affinity maturation is to first diversify the sequences of the antibody fragments and then carry out new rounds of phage display selections to select antibody fragments with increased affinity to the target antigen. Several different methods to achieve diversification exist. One such method is chain shuffling [51], where the antibody fragments obtained after selection either have their heavy or their light chain replaced with the full repertoire of this chain from a naïve library (often the same library from which the initial antibody fragment was selected) (Figure 9A). Thereby, a new phage display antibody library is created with a constant heavy or light chain and the full range of the opposite chain as partners (i.e., one heavy chain combined with all available light chains, or one light chain combined with all available heavy chains). Phage display selections are then conducted again to identify the best match between heavy and light chains, which often results in antibody fragments with increased affinity to their targets. Other methods of diversification used for in vitro affinity maturation employ mutagenesis of the variable regions of the antibodies. These mutations can either be introduced through random mutagenesis of the whole variable region of the antibodies [52] (Figure 9B) or through different types of site-directed mutagenesis techniques that specifically target the different complementarity-determining regions (CDRs) in the variable regions of the antibodies (Figure 9C) [53].

## 9. Selected Examples of the Use of Antibody Phage Display Selection within Toxinology

Phage display selection has been employed in the field of toxinology to obtain antibody fragments with specificities to different toxins [3] and was first employed within snakebite antivenom research in 1995 by Meng et al., who used a murine scFv library to discover antibody fragments against crotoxin from *Crotalus durissus terrificus* [54]. The employed scFv library was affinity matured as it was generated from mice that had been immunized with crotoxin. The dissociation constant between one obtained scFv and crotoxin was determined to be 7.0 × 10^−10^ M, reflecting a very strong binding interaction between the antibody fragment and the target toxin. The antibody fragment was tested for its ability to inhibit phospholipase A_2_ activity in vitro without any effects even at 5:1 antibody-to-toxin molar ratios. However, the scFv successfully delayed death and in some cases even prevented death in mice injected with lethal doses of Mojave toxin (a crotoxin homolog) pre-incubated with the scFv [54].

Since 1995, phage display technology has been employed using different target toxins from different animal species (including snake, scorpion, spider, and bee toxins), using libraries containing murine, human, and camelid antibodies [2,12]. As an example, Kulkeaw et al. were the first to use a human antibody phage display library [55]. This library consisted of human svFvs and was used to discover antibody fragments against α-cobratoxin (a long chain α-neurotoxin) from *Naja kaouthia*. The scFvs selected from this library were tested for their ability to neutralize one LD_100_ of α-cobratoxin upon pre-incubation of each scFv and the toxin in vivo. This experiment demonstrated that the selected human scFvs mainly provided prolonged survival of the mice and failed to protect them from lethality. However, the best scFv was able to protect 33% (2/6) of the mice administered with scFv and toxin from death. In comparison, Richard et al. employed a llama V_H_H phage display library to discover antibody fragments against α-cobratoxin from *N. kaouthia* [7]. Here, the library was affinity matured as it was based on V_H_H genes from a llama immunized with *N. kaouthia* crude venom. The obtained antibody fragments had dissociation constants as low as 4 × 10^−10^ M and proved effective both in protecting and rescuing mice in a lethality study at antibody-to-toxin molar ratios as low as 0.75:1.

Several examples of phage display selection being employed for discovery of antibody fragments with specificity to scorpion toxins can be found in the literature. One such example was reported by Rodríguez-Rodríguez et al., where a human scFv library was used to discover scFvs against Mexican scorpion venom toxins [56]. Here, scFvs with the ability to bind Cn2 toxin (a β-neurotoxin) from *Centruroides noxius* were discovered. However, the scFvs were not able to neutralize the Cn2 toxin in vivo. To improve the affinity of the antibody fragments, in vitro affinity maturation methods involving directed evolution, site-directed mutagenesis, and random mutagenesis were employed. As a result, the researchers successfully improved an scFv to become effective in protecting mice from both the Cn2 toxin from *C. noxius*, the Cll1 toxin from *Centruroides limpidus*, the Css2 toxin from *Centruroides suffusus*, and whole venom from *C. suffuses* [56]. Another example is provided by Pucca et al., where the human scFv library, Griffin.1, was employed to discover scFvs against *Tityus serrulatus* toxins [57]. One obtained scFv was investigated for its neutralizing abilities both through in vitro biochemical assays and in a murine model, where it showed partially neutralizing effects. In a later study of the same scFv, it was additionally shown that the scFv could neutralize the effects of similar β-neurotoxins from other scorpion genera [58].

Finally, phage display selection has also been employed to discover antibody fragments against bee venom toxins [59]. In a study by Pessenda et al., the human scFv library, Griffin.1, was used to find scFvs against melittin and phospholipase A_2_ from the Africanized bee *Apis mellifera*. These scFvs were shown to inhibit the hemolytic activity of the venom in vitro and displayed the ability to reduce the edematogenic activity when the scFvs were pre-incubated with the venom and injected into the paws of mice. Additionally, prolongation of survival was observed when mice were subjected to lethal doses of venom pre-incubated with the scFvs. However, full protection against lethality was not observed [59].

In relation to toxin-targeting, it is important to note that although antibodies with specific toxin-binding specificities can often easily be discovered using phage display selection, such binding does not necessarily translate into toxin-neutralizing ability in vivo [60,61,62]. Different modes of toxin neutralization likely exist [63], and any phage display selection experiment must be followed by vigorous testing of functional activity of the isolated toxin-binding antibodies, as antibodies binding to non-neutralizing epitopes may have limited therapeutic value.

## 10. Closing Remarks

With the renewed international focus on snakebite envenoming, more focus on innovation and development within the field of antivenom will likely arise. Phage display technology represents a robust, inexpensive, and easy to perform discovery approach that is particularly suitable for large-scale antibody discovery projects within antivenom research. In this area, multiple antibodies against a vast range of different toxin targets need to be developed, and phage display selection offers the opportunity to do so in a parallelized manner. Although no other animal envenomings than snakebite are currently included in the World Health Organization’s list of Neglected Tropical Diseases, these analogous fields may likely benefit from the inclusion of snakebite envenoming on this list. The use of phage display technology within toxinology and antivenom research is still at its infancy. However, several promising examples have already been reported in the literature, supporting the notion that phage display selection is indeed a feasible antibody discovery approach. Additionally, the many developments and advances within antibody technologies and manufacturing, championed by other fields, such as oncology and autoimmune and infectious diseases, represent a golden opportunity that antivenom research may piggy-back on. It therefore seems relevant that antivenom researchers become acquainted with phage display technology as well as other antibody discovery approaches.

## Figures and Tables

**Table 1:** Gene name, protein name, protein size, and the function of the genes carried by the M13 phage [16].

Gene Name	Protein Name (Abbreviation)	Size (kDa)	Function
***I***	Gene 1 protein (G1P)	39.6	Assembly
	Gene 11 protein (G11P)	12.4	Assembly
***II***	Replication-associated protein (G2P)	46.2	Replication
	Gene 10 protein (G10P)	12.7	Replication
***III***	Attachment protein (G3P)	44.7	Coat protein Adsorption and extrusion
***IV***	Virion export protein (G4P)	45.9	Assembly and extrusion
***V***	DNA-binding protein (G5P)	9.7	Replication
***VI***	Head virion protein (G6P)	12.4	Coat protein Infection and budding
***VII***	Tail virion protein (G7P)	3.6	Coat protein Assembly and budding
***VIII***	Capsid protein (G8P)	7.6	Coat protein
***IX***	Tail virion protein (G9P)	3.7	Coat protein Assembly and budding

**Table 2:** Genetic elements carried by phagemids and helper phages.

Genetic Element	Phagemid	Helper Phage
Gene *I*		X
Gene *II*		X
Gene *III*		X
Gene *IV*		X
Gene *V*		X
Gene *VI*		X
Gene *VII*		X
Gene *VIII*		X
Gene *IX*		X
Gene *III-scFv*	X	
Antibiotic resistance gene	X	X
Origin of replication	X	
Origin of replication (inefficient)		X
